# Emerging Multimodal Point-of-Care Diagnostic Strategies for Rapid Detection and Management of Respiratory Viruses: A State-of-the-Art Review

**DOI:** 10.3390/diagnostics16132048

**Published:** 2026-06-30

**Authors:** Helal F. Hetta, Abdul Haseeb, Salwa Qasim Bukhari, Zinab Alatawi, Ahmad J. Mahrous, Mahmoud E. Elrggal, Mohammad Al Masri, Ahmed A. Kotb

**Affiliations:** 1 Division of Microbiology, Immunology and Biotechnology, Department of Natural Products and Alternative Medicine, Faculty of Pharmacy, University of Tabuk, Tabuk 71491, Saudi Arabia; 2 Department of Pharmacy Practice, Faculty of Pharmacy, University of Tabuk, Tabuk 71491, Saudi Arabia; ahanif@ut.edu.sa; 3 Department of Diagnostic Radiology, Faculty of Medicine, University of Tabuk, Tabuk 71491, Saudi Arabia; s.bukhari@ut.edu.sa; 4 Department of Family and Community Medicine, Faculty of Medicine, University of Tabuk, Tabuk 47512, Saudi Arabia; zalatawi@ut.edu.sa; 5 Department of Pharmaceutical Practices, College of Pharmacy, Umm Al-Qura University, Makkah 21955, Saudi Arabia; ajmahrous@uqu.edu.sa; 6 College of Medicine, Al Qunfudah Umm Al-Qura University, Al Qunfudhah 28821, Saudi Arabia; merggal@uqu.edu.sa; 7 Faculty of Allied Medical Sciences, Hourani Center for Applied Scientific Research, Al-Ahliyya Amman University, Amman 19111, Jordan; masri@ammanu.edu.jo; 8 Department of Microbiology and Immunology, Faculty of Pharmacy, Assiut University, Assiut 71515, Egypt; ahmedabedelaziz994@aun.edu.eg

**Keywords:** point-of-care testing (POCT), respiratory viruses, molecular diagnostics, isothermal amplification, internet-of-things (IoT), smartphone-based diagnostics, resource-limited settings, multiplex nucleic acid testing

## Abstract

The co-circulation of respiratory viruses, including SARS-CoV-2, influenza A/B, and respiratory syncytial virus (RSV), represents a significant global health challenge that requires rapid, accurate, and differential diagnosis to support infection control and appropriate clinical decision-making. This narrative review summarizes emerging multimodal point-of-care testing (POCT) strategies for the detection and management of these respiratory viruses. Relevant studies were identified through literature searches of major scientific databases, including PubMed, Scopus, and Web of Science, focusing on recent advances in molecular diagnostics, biosensors, microfluidics, and digital health technologies. To improve clinical interpretation and comparative assessment, current POCT platforms were organized into four operational tiers based on infrastructure dependence, degree of portability, and level of decentralization of testing. Tier 1 (Professional Clinical Systems) includes fully integrated automated molecular diagnostic platforms designed for use in hospital and emergency care settings. Tier 2 (Field-Deployable Systems) comprises portable molecular and isothermal amplification technologies designed for use in decentralized or resource-limited environments. Tier 3 (Hardware-Lite Assays) includes simplified diagnostic approaches that minimize instrument requirements and are suitable for near-patient or low-infrastructure settings. Tier 4 (Consumer-Digital Diagnostics) encompasses emerging smartphone- and IoT-integrated diagnostic platforms that support user-driven testing and digital health connectivity. This tier-based framework reflects a proposed stratification of POCT technologies along a decentralization continuum and aims to facilitate comparison and selection of diagnostic strategies across diverse healthcare settings.

## 1. Introduction

Respiratory viruses such as SARS-CoV-2, influenza A virus, influenza B virus, and respiratory syncytial virus (RSV) continue to co-circulate globally, producing overlapping clinical presentations that complicate timely treatment decisions, antimicrobial stewardship, and infection control. The COVID-19 pandemic accelerated the decentralization of molecular diagnostics from centralized laboratories toward near-patient and point-of-care environments, where rapid differentiation between viral etiologies became increasingly important for triage and clinical decision-making [1,2,3,4,5].

Despite this expansion, the current point-of-care testing (POCT) landscape remains highly heterogeneous, with substantial variation in infrastructure requirements, analytical performance, workflow integration, and intended use settings. This heterogeneity complicates the selection of appropriate diagnostic solutions when decisions are based solely on analytical speed or sensitivity without considering operational context [6,7,8,9,10,11].

Traditional POCT classifications are primarily based on analytical methodology (e.g., PCR, isothermal amplification, lateral flow assays), which does not fully capture differences in deployment conditions, user requirements, and system integration. Recent studies highlight that modern POCT platforms differ not only in molecular chemistry but also in portability, automation level, reagent stability, and digital connectivity [8,10,12,13].

Existing conceptual frameworks, including ASSURED and REASSURED criteria, address important aspects of diagnostic quality and implementation; however, a structured operational synthesis specifically focused on respiratory POCT technologies remains limited. Therefore, there is a need for a context-oriented framework that integrates diverse diagnostic platforms into a unified operational structure while remaining consistent with established decentralization principles [8,11,14,15,16,17,18].

Contemporary respiratory POCT systems can be conceptualized as a proposed operational continuum ranging from fully automated laboratory-based systems to highly decentralized, user-operated platforms. Cartridge-based PCR systems used in clinical settings prioritize automation and rapid turnaround time to support hospital workflows. In contrast, portable microfluidic and isothermal amplification systems are designed to reduce infrastructure dependence and enable deployment in resource-limited environments. Hardware-lite biosensing approaches further minimize instrumentation requirements, while smartphone- and IoT-integrated systems extend diagnostics into community and home-based settings [8,11,16,19,20,21,22].

These design differences reflect distinct engineering trade-offs between analytical performance, portability, cost, and connectivity. Accordingly, grouping POCT technologies solely by molecular method is insufficient to describe their operational behavior across real-world settings. Building on established decentralization principles, a tiered operational perspective is proposed here as a practical framework for comparing diagnostic platforms according to their deployment characteristics and intended use environments [10,23,24,25,26].

In this context, the present review proposes a four-tier operational framework for respiratory POCT, comprising: (i) professional clinical systems, (ii) field-deployable systems, (iii) hardware-lite assays, and (iv) consumer-digital diagnostics. This framework is informed by recent advances in molecular diagnostics, biosensors, microfluidics, and digital health technologies, and aims to support alignment between diagnostic architecture, clinical setting, and public health needs.

## 2. Methods for Literature Review and Evidence Selection

A structured narrative review was conducted to identify peer-reviewed studies on point-of-care testing (POCT) technologies for respiratory viruses, including SARS-CoV-2, influenza viruses, and respiratory syncytial virus (RSV). Literature searches were performed in PubMed, Scopus, and Web of Science for studies published between January 2015 and February 2026.

Search strategies combined controlled vocabulary and free-text terms, including “point-of-care testing”, “molecular diagnostics”, “respiratory pathogens”, “isothermal amplification”, “microfluidics”, “CRISPR diagnostics”, and “smartphone-based diagnostics”, using Boolean operators (“AND” and “OR”). In addition, reference lists of included articles were manually screened to identify further relevant studies.

Studies were eligible for inclusion if they described POCT or near-patient diagnostic technologies for respiratory viruses and reported analytical or clinical performance characteristics. Exclusion criteria included editorials, conference abstracts, duplicate publications, non-peer-reviewed sources, and studies not focused on respiratory POCT applications or lacking sufficient methodological information for extraction.

Data extraction included diagnostic principle, assay format, reported analytical performance, sample type, level of portability, infrastructure requirements, and intended deployment context. Extracted information was synthesized narratively due to heterogeneity in study designs, diagnostic platforms, and reported performance metrics, which precluded formal meta-analysis.

Technologies were subsequently organized into four operational tiers based on reported infrastructure dependence, degree of automation, portability, and level of digital integration. This categorization was applied as an interpretive framework for comparative analysis rather than a formal statistical classification system.

This review follows a structured narrative approach intended to provide a comprehensive overview of emerging respiratory POCT technologies, rather than a systematic evidence synthesis.

It is acknowledged that the included technologies represent different stages of development and clinical validation. Established commercial platforms, such as cartridge-based molecular systems, have undergone extensive clinical evaluation and real-world deployment, whereas several emerging technologies remain at the laboratory validation or pilot study stage. Early-stage platforms, including prototype biosensors and digitally integrated diagnostic systems, are included to highlight emerging technological directions and translational potential rather than to imply equivalent clinical readiness across all platforms.

## 3. Tier 1 (Professional Clinical)

Tier 1 point-of-care platforms are designed for rapid molecular diagnosis in acute clinical settings such as emergency departments and hospital wards. These systems typically provide turnaround times below 30 min and use enclosed cartridge-based workflows with minimal operator intervention. Their primary objective is to support rapid triage, antimicrobial stewardship, and timely clinical decision-making.

**Figure 1:**
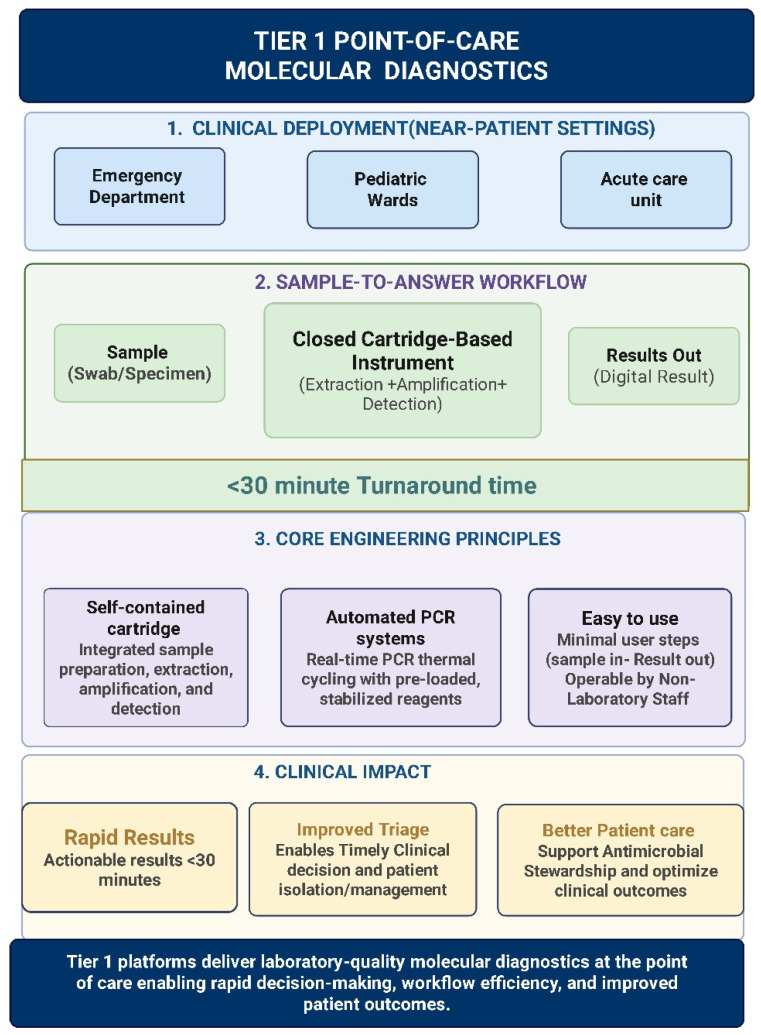
Tier 1 point-of-care molecular diagnostics framework. Near-patient deployment (e.g., emergency departments, pediatric wards, acute care units) using closed, cartridge-based systems that integrate extraction, amplification, and detection in a fully automated workflow. These platforms deliver digital results in <30 min, leveraging self-contained cartridges, automated real-time PCR with preloaded reagents, and minimal user steps suitable for non-laboratory staff, enabling rapid triage, timely clinical decision-making, and improved patient outcomes (created with BioRender.com).

Technologically, Tier 1 platforms (Figure 1) achieve this performance through three core engineering principles: 1. Closed, self-contained cartridges integrating nucleic acid extraction, amplification, and detection. 2. Automated real-time PCR thermal cycling with pre-loaded, stabilized reagents. 3. Minimal user steps (sample in → result out) to allow operation by non-laboratory staff.

This design philosophy facilitates the deployment of molecular diagnostics from centralized laboratories to bedside or near-patient settings without sacrificing analytical performance. Clinical evidence demonstrates that these systems provide near-laboratory RT-PCR accuracy while reducing the time to actionable treatment decisions. For example, the BioFire SPOTFIRE R/ST Panel detects 15 respiratory and bacterial targets from nasopharyngeal or throat swabs within 17 min, showing 92–100% agreement with reference laboratory methods. Such speed may facilitate real-time triage decisions in crowded emergency departments and supports immediate isolation or treatment pathways [2]. Positive percent agreement is 100% while negative percent agreement is 92–100%.

Similarly, cost–consequence modeling in primary care has shown that the Xpert Xpress CoV-2/Flu/RSV Plus platform yields the highest number of appropriate treatment decisions compared with antigen testing, empiric diagnosis, and send-out PCR, while also reducing overall healthcare costs by preventing delayed or inappropriate therapy. Using Xpert Xpress at POC combined fast turnaround with high diagnostic accuracy, thereby increasing correct treatment courses while reducing total costs for influenza [27].

The iNAT SARS-CoV-2/Flu A/Flu B/RSV Assay, a multiplex reverse transcription quantitative PCR (RT-qPCR) point-of-care testing (POCT) system, demonstrated limits of detection of 45 copies/mL for SARS-CoV-2, 133 copies/mL for influenza A, 57 copies/mL for influenza B, and 212.5 copies/mL for RSV. These performance characteristics are supported by multiplex fluorescence channel optimization and rapid thermal cycling, with a turnaround time of 30 min. In clinical validation, the assay showed 99.36% agreement with the National Medical Products Administration (NMPA)-approved reference method [28].

Importantly, Tier 1 systems have also demonstrated behavioral and stewardship impact. Introduction of the Enigma MiniLab FluAB-RSV, a rapid POCT for influenza A/B and RSV infections, in pediatric wards significantly increased appropriate oseltamivir prescribing and reduced reimbursement costs without prolonging hospital stay, highlighting how rapid molecular results directly influence prescribing practices and decrease laboratory costs [29].

Longitudinal deployment of the Cepheid Xpert Xpress in emergency departments has supported surveillance programs by enabling monitoring of asymptomatic viral carriage, nosocomial introduction risk, and seasonal re-emergence of influenza and RSV following COVID-19 containment periods [30].

Alere i influenza A & B is a near-patient, cartridge-based molecular diagnostic assay that detects influenza A and B viruses using nicking enzyme amplification reaction (NEAR), an isothermal nucleic acid amplification technology that removes the need for thermal cycling and enables rapid sample-to-result processing. The test is performed directly from a nasopharyngeal swab inserted into a single-use cartridge, delivering results within approximately 13–15 min through a fully integrated and simplified workflow suitable for point-of-care settings. Clinical performance studies have shown a low limit of detection in the range of a few hundred viral RNA copies per reaction, resulting in substantially higher sensitivity than conventional rapid antigen tests and approaching that of standard RT-PCR assays, although diagnostic performance may vary depending on viral load and study conditions. Overall, its combination of rapid turnaround time, ease of use, and high analytical accuracy has supported its adoption in emergency departments and urgent care environments, where it facilitates timely antiviral therapy decisions and enhances infection control measures during influenza outbreaks [31,32].

The Film Array Respiratory Panel 2 (RP2) is a fully automated multiplex PCR assay that detects 22 respiratory pathogens directly from nasopharyngeal swabs within approximately 45 min. It represents an improved version of the earlier Film Array RP (1.7), with redesigned assays, expanded pathogen coverage, and reduced run time. The panel identifies a broad spectrum of viral and atypical bacterial agents, including influenza A and B subtypes, RSV, parainfluenza viruses, adenoviruses, endemic coronaviruses, rhinovirus/enterovirus, human metapneumovirus, and key bacterial pathogens such as *Bordetella pertussis*, *Bordetella parapertussis*, *Chlamydia pneumoniae*, and *Mycoplasma pneumoniae*, as well as emerging targets such as MERS-CoV. In a multicenter evaluation of 1612 nasopharyngeal samples compared with reference PCR and sequencing, RP2 showed high overall agreement (99.2%). Positive percent agreement was ≥91.7% for most targets, and negative percent agreement was ≥93.8% across all analytes. Slightly lower agreement was observed for a few organisms, including coronavirus OC43 and *Bordetella* species, while adenovirus detection showed improved sensitivity due to broader genotype coverage. Overall, RP2 demonstrated high diagnostic accuracy and a significantly reduced turnaround time, supporting its value in rapid respiratory infection diagnosis across clinical settings [33]. The BioFire Film Array Respiratory Panel 2.1 (RP2.1) extends this platform by adding SARS-CoV-2, enabling simultaneous detection of COVID-19 with other respiratory pathogens during overlapping seasonal outbreaks. Multicenter studies showed pathogen detection in about 19% of samples, with SARS-CoV-2 identified in 12.6%. SARS-CoV-2 performance was highly concordant with reference methods, with ~98% positive and ~99% negative percent agreement, and most discrepancies occurring near the limit of detection. Rhinovirus/enterovirus was more frequent in pediatric populations, while SARS-CoV-2 predominated in adults. This expanded panel improves etiological differentiation in clinically similar respiratory infections and supports timely clinical decision-making [33,34].

The BioFire Film Array Pneumonia Panel is a syndromic multiplex PCR assay developed for rapid identification of lower respiratory tract pathogens, including bacteria, viruses, and antimicrobial resistance markers, directly from sputum and bronchoalveolar lavage (BAL) specimens, with an approximate turnaround time of one hour. Across clinical validation studies, it has demonstrated high analytical performance compared with standard culture-based diagnostics. In a multicenter ICU study involving BAL and sputum samples, the panel achieved high sensitivity (~96–100%) and specificity (~90–98%), varying by specimen type and pathogen group, with particularly robust detection of common bacterial and viral pathogens and high concordance for resistance gene identification (positive percent agreement ~95–97%) when benchmarked against conventional susceptibility testing.

However, real-world evidence has highlighted important interpretative challenges. In a large cohort of hospitalized patients with BAL samples, the assay significantly increased pathogen detection rates (36% versus 16% with culture), but not all positive results corresponded to clinically confirmed pneumonia, reflecting the potential influence of airway colonization rather than true infection. Additional multicenter findings support this limitation, emphasizing that molecular detection may also identify non-viable organisms or commensal flora, necessitating interpretation alongside clinical and radiological findings. Overall, despite these considerations, the panel substantially improves diagnostic yield and reduces time to pathogen identification, thereby enhancing antimicrobial stewardship and supporting faster, more informed decision-making in intensive care and hospital-acquired pneumonia management [33,35,36].

The QIAstat-Dx SARS-CoV-2 panel is a cartridge-based multiplex real-time PCR assay capable of detecting SARS-CoV-2 alongside a broad range of up to 17 respiratory viruses, supporting a syndromic diagnostic approach for patients with acute respiratory symptoms. In an evaluation of 120 archived clinical respiratory specimens, the assay demonstrated high diagnostic performance for SARS-CoV-2 detection, with a sensitivity of 90.0% and specificity of 100%. The positive predictive value reached 100%, while the negative predictive value and overall accuracy were both approximately 99.9%, indicating consistent analytical performance for clinical application.

In a subset analysis involving 27 specimens tested for additional respiratory viruses, results showed moderate concordance (77.7%) when compared with the BioFire FilmArray RP1.7 platform, suggesting variability in detection agreement across multiplex targets. Despite its advantages in rapid turnaround time, minimal operator requirement, and high analytical accuracy, limitations include high operational cost and reduced performance at higher cycle threshold values (Ct > 35), which may affect sensitivity in low-viral-load samples [37].

The ePlex Respiratory Pathogen (RP) panel (GenMark Diagnostics) is a fully automated, sample-to-answer multiplex PCR assay designed for the simultaneous detection of a broad spectrum of respiratory pathogens, including 19 viruses (influenza A and its subtypes, influenza B, adenovirus, endemic coronaviruses, rhinovirus/enterovirus, human metapneumovirus, parainfluenza viruses 1–4, and RSV A/B) as well as two atypical bacteria (*Mycoplasma pneumoniae* and *Chlamydia pneumoniae*). The assay enables rapid molecular diagnosis directly from nasopharyngeal swab specimens and is positioned for use in acute respiratory infection workflows where timely pathogen identification is critical.

In a large multicenter evaluation including 2908 prospectively and retrospectively collected specimens, the ePlex RP panel was compared with the BioFire FilmArray Respiratory Panel as the reference method. Overall agreement exceeded 95% across all analytes, demonstrating strong concordance between platforms. Positive percent agreement for viral targets ranged from 85.1% to 95.1%, while negative percent agreement remained consistently high (99.5–99.8%), indicating excellent specificity. Discordant analysis of 12% of samples using targeted PCR and sequencing confirmed a proportion of ePlex-positive results, supporting its analytical reliability. Reproducibility testing showed 100% agreement for most pathogens, with slightly reduced performance for adenovirus at low viral concentrations, which improved at higher viral loads. Overall, the ePlex RP panel demonstrates high diagnostic accuracy, high reproducibility, and rapid turnaround time, making it a multiplex diagnostic platform for the detection of common viral and atypical bacterial respiratory pathogens. Its performance characteristics support its integration into clinical respiratory testing algorithms to facilitate timely diagnosis, improve patient management, and enhance infection control strategies in acute care settings [38].

Collectively, these examples demonstrate that Tier 1 platforms are characterized not merely by speed, but by clinical integration: they function as decision-support tools embedded within hospital workflows. The primary limitation of Tier 1 systems is instrument cost and infrastructure dependence, which restricts their use to well-resourced clinical environments. This limitation directly motivates the need for Tier 2 and Tier 3 solutions, where portability and independence from complex instrumentation become the dominant design drivers.

Despite their good analytical performance and rapid turnaround times, Tier 1 systems remain associated with several operational limitations, including high instrument cost, dependence on proprietary cartridges, maintenance requirements, and limited accessibility in low- and middle-income settings. Although false-positive and false-negative rates are generally low, assay performance may decline in samples with low viral load or near the limit of detection. In addition, the closed-cartridge architecture reduces contamination risk but increases per-test cost and dependence on commercial supply chains. These factors may limit scalability outside well-resourced healthcare systems.

## 4. Tier 2 (Field-Deployable)

**Figure 2:**
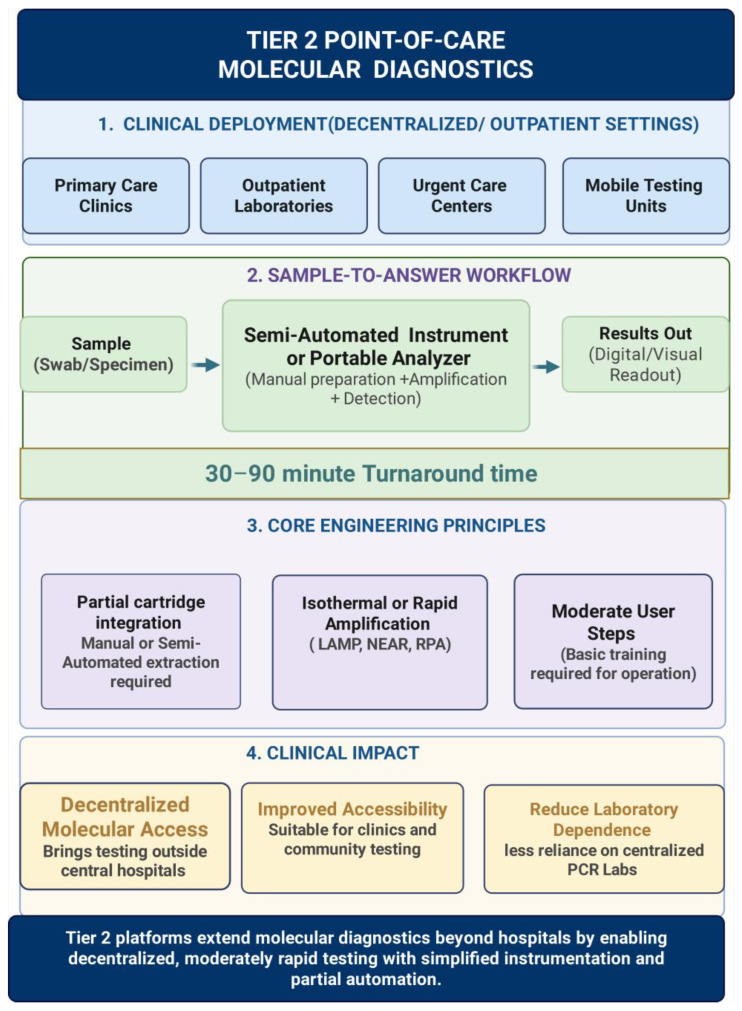
Tier 2 point-of-care molecular diagnostics framework. Decentralized deployment across primary care, outpatient labs, urgent care, and mobile units; a semi-automated sample-to-answer workflow (manual prep with amplification/detection) delivering results in ~30–90 min; core design features including partial cartridge integration, isothermal/rapid amplification (e.g., LAMP, NEAR, RPA), and moderate user steps; enabling expanded access, improved testing availability, and reduced reliance on centralized PCR laboratories (created with BioRender.com).

Tier 2 platforms are defined by portability, reagent stability without cold chain, and high analytical performance outside conventional laboratories. Unlike Tier 1 systems that optimize hospital workflow through automation, Tier 2 systems (Figure 2) are engineered to operate reliably in remote clinics, mobile units, outbreak zones, and resource-limited settings where electricity, refrigeration, and trained personnel may be constrained.

From an engineering perspective, Tier 2 technologies converge on four enabling strategies: 1. Lyophilized, pre-stored reagents to eliminate refrigeration needs. 2. Integrated microfluidics for automated fluid handling without complex user steps. 3. Isothermal or rapid PCR chemistries to reduce power and thermal cycling demands. 4. Compact, battery-operated or low-power controllers for true field use. These principles allow Tier 2 devices to deliver near-Tier-1 molecular accuracy while removing the infrastructure barriers that limit hospital-grade platforms.

Evidence for this design philosophy is demonstrated by field multiplex PCR systems capable of detecting up to 14 respiratory viral and bacterial pathogens, including SARS-CoV-2, influenza A virus, influenza B virus, RSV, human rhinovirus, adenovirus, human metapneumovirus (hMPV), *Streptococcus pneumoniae*, *Klebsiella pneumoniae*, *Bordetella pertussis*, *Haemophilus influenzae*, *Legionella pneumophila*, *Mycoplasma pneumoniae*, and *Chlamydia pneumoniae*, within 30 min under portable conditions with >97% total accuracy and high reproducibility under portable conditions.

The system demonstrated a limit of detection of 10^3^ copies/mL for most targets. In clinical evaluation, it achieved a positive predictive value (PPV) of 100%, negative predictive value (NPV) of 94.92%, positive percent agreement (PPA) of 93.62%, negative percent agreement (NPA) of 100%, and overall accuracy of 97.09%, with a kappa value of 0.94, indicating high reliability [39]. Collectively, these systems demonstrate that comprehensive syndromic respiratory panels are feasible outside centralized laboratories when reagent stabilization is combined with portable thermocycler platforms.

A more advanced embodiment of Tier 2 is the IoT microfluidic RT-LAMP chip, where RNA extraction, amplification, and fluorescence detection are integrated onto a self-contained chip preloaded with lyophilized buffers and RT-LAMP reagents. This Raspberry Pi-based platform automates valve and heater control to perform molecular testing without external laboratory equipment, identifying SARS-CoV-2 and influenza viruses from clinical respiratory samples within 70 min. The IoT-enabled device integrates a CMOS sensor for fluorescence detection, a resistive heater for temperature regulation, and solenoid valves to control on-chip reagent flow, with a touchscreen interface for operation and result display. Evaluation using 11 clinical samples (five SARS-CoV-2, two influenza A, and four influenza B) demonstrated accurate, multiplex detection with high specificity. The integration of the microfluidic chip with this portable system offers a cost-effective, scalable solution for rapid molecular diagnostics in resource-limited settings [40].

Further simplification is seen in the Passive CRISPR microfluidic chip, which removes pumps and electronics by exploiting capillary action and gravity-driven flow. By combining reverse transcription recombinase polymerase amplification (RT-RPA) with CRISPR-Cas12a detection and lyophilized reagents, the chip enables multiplex viral detection in ~45 min using only a heating block [41]. This illustrates how fluid physics can replace mechanical instrumentation in field diagnostics. By utilizing a rapid, 10-min sample preparation protocol and a 35-min on-chip assay, this platform enables the multiplex detection of influenza A/B, human parainfluenza virus, and SARS-CoV-2. The assay demonstrated a detection sensitivity of about 10 copies/µL for viral RNA in dilution series experiments. The sensitivity of the assay was 98.44% (95% CI: 91.6–99.96%), and the specificity was 100% (95% CI: 79.4–100%) [42].

Portable multiplex RT-PCR devices demonstrate that conventional RT-PCR sensitivity can be preserved in suitcase-sized platforms capable of detecting up to 14 respiratory viruses directly from nasopharyngeal swabs while minimizing pre-analytical steps. The assay targeted influenza A and B, parainfluenza viruses 1–4, hMPV, adenovirus, human rhinovirus, RSV, and SARS-CoV-2. Primer–probe sets were designed for each virus, and standard curves established the limit of detection. Field applicability was assessed using nasopharyngeal samples, with successful detection in most cases on a portable point-of-care device [43,44].

The defining trade-off of Tier 2 is a slightly longer turnaround time in exchange for environmental robustness and infrastructure independence. This trade-off is essential for extending high-quality molecular diagnostics to locations where Tier 1 systems cannot operate.

Tier 2 therefore acts as the bridge between hospital-grade molecular testing and equipment-free visual diagnostics, paving the way toward Tier 3 solutions where instrumentation is minimized even further.

Although Tier 2 platforms improve portability and decentralization, implementation challenges remain. Several systems have been validated using relatively small clinical cohorts, and large multicenter evaluations are still limited for many emerging devices. Environmental factors, reagent stability under extreme field conditions, battery dependence, and variability in operator training may influence reproducibility and diagnostic consistency. Furthermore, while lyophilized reagents improve shelf life and cold-chain independence, long-term storage stability and regulatory standardization remain incompletely evaluated for many platforms.

## 5. Tier 3 (Hardware-Lite)

**Figure 3:**
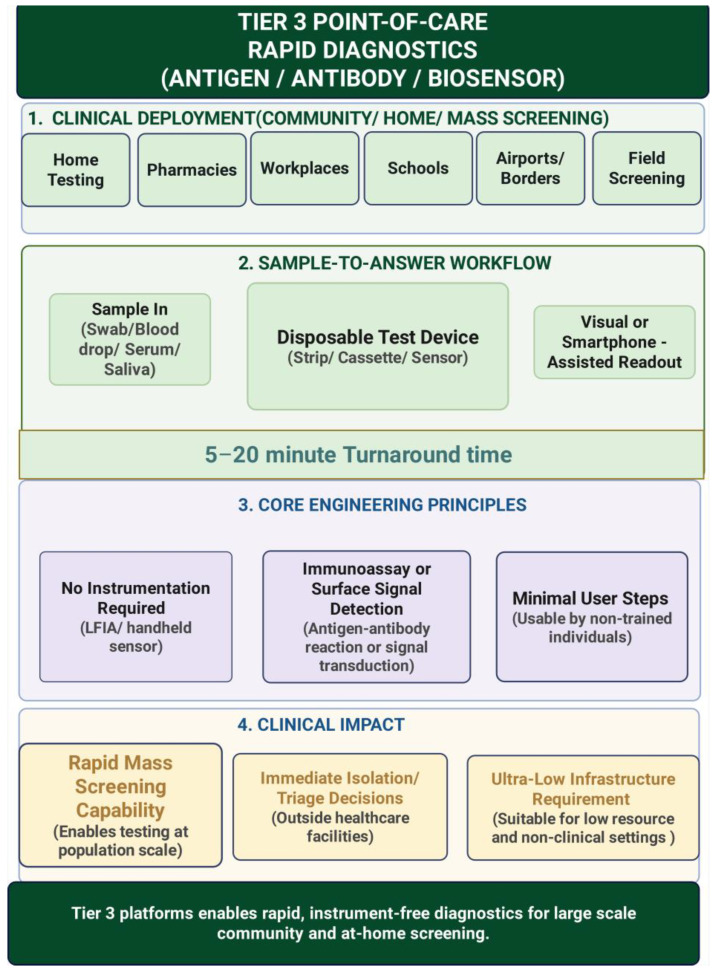
Tier 3 point-of-care rapid diagnostics framework. Community- and home-based deployment (e.g., pharmacies, workplaces, schools, airports, and field settings) utilizing disposable test devices such as lateral flow assays or biosensors. The simplified sample-to-answer workflow (swab, blood, serum, or saliva to visual or smartphone-assisted readout) delivers results in ~5–20 min. These systems require no instrumentation, rely on immunoassay or surface signal detection, and involve minimal user steps, enabling large-scale screening, rapid triage decisions, and use in low-resource or non-clinical environments (created with BioRender.com).

Tier 3 platforms (Figure 3) are defined by minimal hardware dependence, low cost, and visual or simple optical readouts while retaining the molecular specificity of nucleic acid testing. In contrast to Tier 2 systems that miniaturize instrumentation, Tier 3 solutions remove instrumentation altogether or reduce it to a simple heater or handheld reader.

The central engineering shift in Tier 3 is replacing complex instruments with smart chemistry and nanomaterial signal transduction. These platforms rely on three key strategies: 1. Isothermal amplification (e.g., LAMP-like chemistries) to avoid thermocyclers. 2. Nanoparticle or probe-mediated signal conversion to produce visible color or strong optical signals. 3. Lateral flow or strip-based formats to simplify handling and interpretation. This enables true molecular diagnostics in resource-limited clinics, rural settings, and low-skill environments where even portable PCR devices are impractical.

The HiFi-CAMP method advances isothermal diagnostics beyond visual colorimetry by using high-fidelity polymerase-mediated probe cleavage to generate real-time fluorescent signals during amplification. Built on 5′-half-complementary primers (CAMP/HCPA design), the approach combines linear and loop-mediated amplification while enabling probe-based specificity and multiplex capability with only a small heater and simple fluorescence detection. Validated against RT-qPCR, HiFi-CAMP demonstrated sensitivities of 90.0% for SARS-CoV-2, 71.4% for RSV-A, and 78.1% for influenza A, with specificities of 100%, 100%, and 95.5%, and consistencies of 93.0%, 93.3% and 88.2% respectively. A duplex format simultaneously detected RSV-A and SARS-CoV-2. These results highlight how probe-assisted isothermal amplification can achieve high specificity in hardware-light, point-of-care formats suitable for resource-limited settings [45].

For high multiplex capacity without complex electronics, a SERS-nanotag lateral flow microarray encodes distinct nucleic acid targets using surface-enhanced Raman scattering signatures arranged in a 2 × 3 array on a nitrocellulose strip. Signal amplification is achieved through nanophotonics rather than instrumentation, enabling ultrasensitive, quantitative detection on a simple lateral flow format.

The platform enabled simultaneous detection of 11 respiratory pathogens with picomolar limits of detection across a five-order linear dynamic range (1 pM–50 nM). Reported limits of detection ranged from 0.030 to 0.041 pM for targets including influenza A/B, parainfluenza viruses 1–3, RSV, adenovirus, *Coxiella burnetii*, *Legionella pneumophila*, *Chlamydia pneumoniae*, and *Mycoplasma pneumoniae*. These findings demonstrate that nanophotonic encoding on lateral flow strips can support high-throughput, ultrasensitive multiplex detection, highlighting its potential utility for point-of-care respiratory diagnostics [46].

The key trade-off in Tier 3 is analytical power versus simplicity. While these systems may not yet match the multiplex depth or ultra-low limits of detection of Tier 1 platforms, they enable true decentralization of molecular diagnostics by removing dependence on complex devices. Tier 3 therefore represents the chemical and nanotechnological solution to the problem of instrument scarcity, setting the stage for Tier 4 where digital tools, rather than laboratory hardware, become the primary enabler of diagnostics.

The principal advantage of Tier 3 systems is their minimal infrastructure requirement and low operational cost; however, this simplification may come at the expense of reduced multiplex capacity, lower analytical sensitivity, and increased susceptibility to subjective result interpretation in visually read assays. Open-format amplification systems may also carry greater contamination risk compared with enclosed cartridge-based platforms. In addition, many nanotechnology-based assays remain at the proof-of-concept stage and have not yet undergone large-scale clinical validation or commercial standardization.

## 6. Tier 4 (Consumer-Digital)

**Figure 4:**
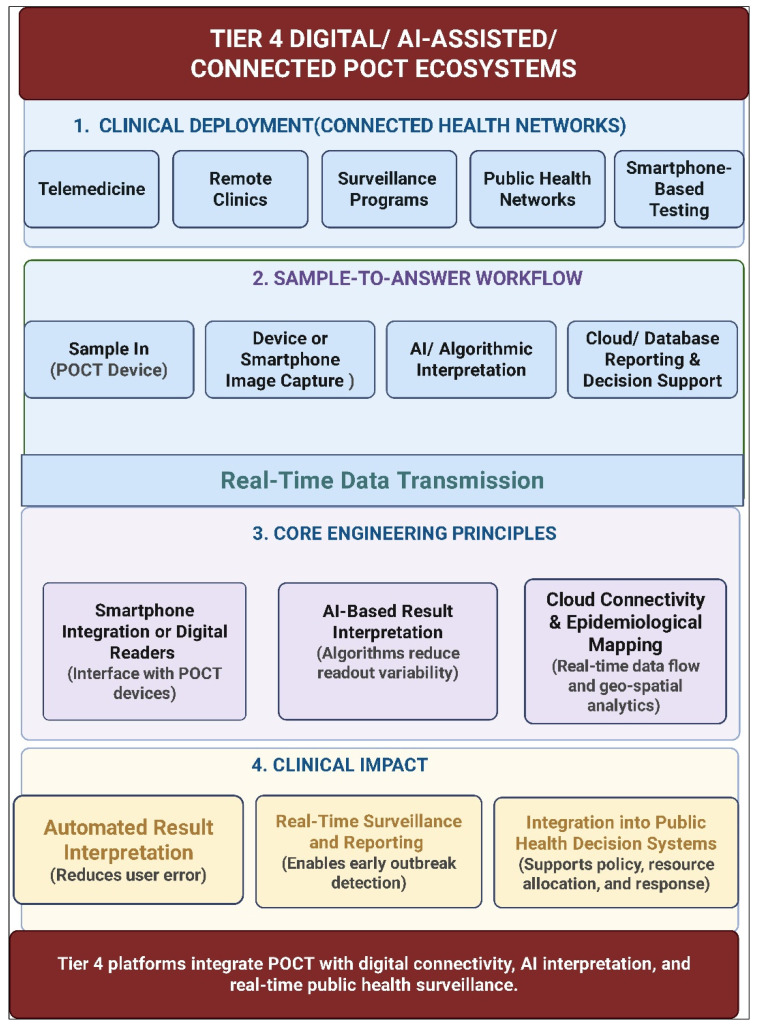
Tier 4 digital/AI-assisted connected POCT ecosystem. Clinical deployment across connected health networks (e.g., telemedicine, remote clinics, surveillance programs, and smartphone-based testing) integrated with a sample-to-answer workflow from POCT device input to smartphone capture, AI-based interpretation, and cloud reporting. Real-time data transmission enables automated result interpretation, reduces user variability, and supports epidemiological mapping. These systems link diagnostics with digital infrastructure to enable real-time surveillance, early outbreak detection, and integration into public health decision-making systems (created with BioRender.com).

Tier 4 (Figure 4) represents a decisive shift in POCT design where consumer electronics and connectivity replace traditional laboratory readers. These systems are intended for home and community settings, transforming molecular tests into digitally connected sensors that support both individual diagnosis and population-level surveillance [47].

The enabling principles of Tier 4 platforms are: 1. Smartphone camera/sensor as the detector for optical or electrochemiluminescent signals. 2. On-chip amplification or direct nucleic acid sensing in compact formats. 3. App-based automated interpretation to remove user subjectivity. 4. Cloud/IoT linkage to aggregate anonymized results for real-time epidemiology. In this architecture, the diagnostic device becomes a data node in a public health network.

A representative example is the Portable Electrochemical Molecular Diagnostic (PEMD) smartphone electrochemiluminescence (ECL) device, a portable electrochemiluminescence platform that uses a LEGO-based heater to maintain LAMP amplification temperatures. A custom smartphone app captures ECL signals and performs automated analysis, enabling compact, field-deployable molecular testing without conventional laboratory hardware. The system integrates a bipolar electrode with a position-resolved ECL sensor chip (B-chip) that connects amplification and detection zones in series, using a DNA-binding redox probe to indicate signal quenching in positive reactions. It simultaneously detected H1N1, H7N9, influenza B, and human adenovirus with a limit of detection of 10 copies/µL. In clinical evaluation, the device correctly identified 87.5% of positive and 91.7% of negative samples, showing strong agreement with qPCR. By combining isothermal amplification, smartphone imaging, and cloud-based analysis, this approach demonstrates proof-of-concept feasibility for multiplex portable molecular testing [48].

A notable shift beyond nucleic acid amplification is illustrated by the Compact Wide-Field Imaging System for High-throughput femtoliter analysis (COWFISH2) platform, which enables amplification-free, multiplex digital RNA detection in a compact format (14 × 3 × 22 cm). By miniaturizing the optics and electronic controls of the original platform and introducing low-cost, durable consumables, the system performs wide-field imaging of femtoliter reaction chambers for direct digital bioanalysis.

Demonstrated in a hospital point-of-care setting, COWFISH2 successfully detected SARS-CoV-2, influenza A, and influenza B RNA without prior amplification. This approach shows how portable digital bioanalysis can be translated to near-patient and community environments, illustrating the potential to expand POCT capabilities beyond conventional amplification-based methods [49].

The central trade-off in Tier 4 is scalability versus regulatory maturity. Widespread adoption depends on robust validation, user-friendly workflows, and data governance. Tier 4 thus extends POCT beyond diagnosis into digital epidemiology. These systems may support broader epidemiological surveillance by enabling integration of anonymized diagnostic data into public health monitoring networks, potentially supporting improved outbreak recognition and public health response [47,50].

In addition to technical validation challenges, Tier 4 consumer-digital POCT systems raise important ethical, regulatory, and implementation considerations. Because many platforms rely on smartphone connectivity, cloud-based data transfer, and app-assisted interpretation, concerns regarding patient privacy, cybersecurity, data ownership, and regulatory compliance become increasingly significant. Protection of sensitive health information and secure transmission of diagnostic data are essential, particularly when integrating POCT results into digital surveillance networks and public health databases. Furthermore, variability in smartphone availability, internet connectivity, digital literacy, and access to mobile health technologies may create inequities in deployment, particularly in low-resource or underserved populations. Although Tier 4 systems offer substantial potential for decentralized surveillance and home-based diagnostics, many amplification-free and consumer-digital platforms remain supported primarily by pilot-scale or laboratory validation studies rather than large multicenter clinical evaluations. Cost-effectiveness, long-term reproducibility, and integration into public health infrastructure also require further investigation before widespread deployment. Collectively, these factors indicate that successful implementation of consumer-digital POCT systems will require not only analytical validation, but also robust regulatory oversight, secure digital infrastructure, and equitable access strategies [15,16,47,50,51,52,53,54,55].

## 7. Cross-Tier Synthesis: Matching Diagnostic Technology to Clinical Environment

The proposed four-tier framework provides a practical approach for aligning respiratory point-of-care testing (POCT) technologies with specific clinical and public health requirements. Rather than identifying a universally optimal diagnostic platform, the framework recognizes that different POCT architectures are designed to address distinct operational needs, deployment settings, and healthcare priorities [16,55].

Current evidence indicates that respiratory POCT technologies inevitably involve trade-offs among diagnostic sensitivity, turnaround time, portability, multiplexing capacity, affordability, and digital connectivity. Consequently, each tier emphasizes different performance characteristics and implementation objectives.

Tier 1 platforms prioritize rapid clinical decision-making, high analytical performance, and integration into hospital workflows. Tier 2 systems extend molecular diagnostic capabilities to field and resource-limited environments through portable and infrastructure-light designs. Tier 3 technologies emphasize affordability, simplicity, and scalability for decentralized testing, whereas Tier 4 platforms expand diagnostic access through digitally connected home-based and community-centered approaches. Accordingly, selection of an appropriate POCT strategy should be guided by intended use, infrastructure availability, user expertise, and clinical urgency rather than analytical performance alone.

The framework also highlights the complementary nature of respiratory POCT technologies. Rather than functioning as competing solutions, the different tiers can operate as components of an integrated diagnostic ecosystem. For example, patients initially assessed using Tier 1 systems in acute-care settings may subsequently be monitored through Tier 4 approaches in the community, while outbreak investigations may utilize Tier 2 platforms for rapid field deployment alongside Tier 3 technologies for broader population screening. This perspective shifts the focus from identifying the “best” POCT platform to determining the most appropriate technology for a given clinical, epidemiological, and infrastructural context.

Importantly, the technologies shown in Table 1 represent different stages of development and clinical readiness. Tier 1 platforms are predominantly commercially available systems supported by regulatory approval and multicenter clinical validation, whereas many Tier 2–4 technologies remain in pilot-stage, laboratory-validation, or proof-of-concept phases. Therefore, comparisons across tiers should be interpreted within the context of technological maturity, intended deployment environment, and regulatory status.

Several limitations should be considered when interpreting the current evidence base. Many emerging POCT platforms have been evaluated primarily in small or single-center studies, limiting generalizability across diverse healthcare settings. Variability in study design, sample preparation methods, contamination-control procedures, reagent stability, and performance-reporting standards further complicates direct comparison between technologies. In addition, implementation challenges—including instrument cost, supply-chain dependence, regulatory approval requirements, interoperability with healthcare information systems, cybersecurity considerations, and unequal access to digital infrastructure—may influence real-world adoption and sustainability, particularly in resource-limited settings. Addressing these challenges will require coordinated efforts among clinicians, engineers, regulatory agencies, public health authorities, and digital health stakeholders [47,56,57].

In conclusion, the proposed four-tier classification offers a context-oriented framework for evaluating and implementing respiratory POCT technologies based on operational deployment characteristics rather than analytical methodology alone. By emphasizing the complementary roles of diverse diagnostic architectures across healthcare environments, the framework supports more informed technology selection and deployment strategies. As respiratory diagnostics continue to evolve, context-driven implementation approaches may provide greater clinical and public health value than attempts to identify a single universally optimal POCT platform.

**Table 1:** Comparison of representative respiratory point-of-care testing (POCT) platforms across the proposed four-tier operational framework according to analytical performance, deployment characteristics, validation status, regulatory readiness, and intended use environment.

Platform	Tier	LoD	Target (s)	TAT (min)	Diagnostic Performance	Validation Status	Regulatory Status	Technology Maturity	Sample Type	Refs.
BioFire SPOTFIRE R/ST	1	Viral: (1.1 × 10^0^–1.8 × 10^4^) copies/mL (strain/pathogen-dependent)	15 Respiratory pathogens (viral & bacterial)	17	PPA/NPA 92–100%	Multicenter validation	FDA-cleared/CE-IVD	Commercially implemented	NP/throat swab	[2,19,58,59,60]
Xpert Xpress CoV-2/Flu/RSV Plus	1	Viral: (2.5 × 10^1^–4.0 × 10^2^) copies/mL	SARS-CoV-2, Flu A/B, RSV	~30	PPA/NPA 95–100%	Clinical implementation	FDA-cleared/CE-IVD	Commercially implemented	NP swab	[27,30,61,62,63]
iNAT SARS-CoV-2/Flu/RSV	1	45–212.5 copies/mL (strain-dependent)	SARS-CoV-2, Flu A/B, RSV	30	99.36% agreement with National Medical Products Administration (NMPA)-approved reference tests	Clinical validation	NR	Early deployment	NP swab	[28]
Alere i Influenza A/B	1	10^2^–10^5^ TCID_50_/mL (strain-dependent)	Influenza A/B	13–15	Sensitivity: 88.8–100%; Specificity: 94.5–100% (vs. culture & RT-PCR)	Multicenter evaluation	FDA-cleared	Commercially implemented	NP swab	[31,32]
FilmArray RP2/RP2.1	1	RSV at 9.0 × 10^0^ copies/mL, Influenza (A & B) from 2.1 × 10^1^ to 3.3 × 10^2^ copies/mL, EnterovirusD68/Rhinovirus from 2.6 × 10^1^ to 3.8 × 10^1^ copies/mL, Coronaviruses (including SARS-CoV-2) from 5.4 × 10^1^ to 2.0 × 10^3^ copies/mL, Parainfluenza (1–4) from 3.0 × 10^1^ to 1.6 × 10^3^ copies/mL, and Adenovirus at 3.0 × 10^3^ copies/mL.	22 respiratory pathogens	45	PPA ≥ 91.7%; NPA ≥ 93.8%	Multicenter validation	FDA-cleared/CE-IVD	Commercially implemented	NP swab	[33,34,64,65]
FilmArray Pneumonia Panel	1	Adenovirus from 1.8 × 10^3^ to 3.5 × 10^4^ copies/mL, Coronavirus from 8.1 × 10^1^ to 1.0 × 10^4^ copies/mL, Human metapneumovirus at 5.9 × 10^3^ copies/mL, Human rhinovirus/enterovirus from 5.7 × 10^2^ to 6.6 × 10^3^ copies/mL, Influenza (A & B) from 2.1 × 10^2^ to 1.7 × 10^3^ copies/mL, Parainfluenza virus from 3.8 × 10^2^ to 8.1 × 10^3^ copies/mL, and Respiratory syncytial virus at 4.3 × 10^2^ copies/mL.	Respiratory bacteria, viruses, AMR markers	60	Sensitivity 96–100%; Specificity 90–98%	Multicenter studies	FDA-cleared/CE-IVD	Commercially implemented	BAL/sputum	[33,35,36,66]
QIAstat-Dx	1	SARS-CoV-2: 500–1000 copies/mL (strain dependent)	SARS-CoV-2 + respiratory viruses	~60	Sensitivity 90%; Specificity 100%	Clinical validation	FDA-cleared/CE-IVD	Commercially implemented	NP swab	[37,67,68,69]
ePlex RP	1	TCID_50_/mL: CoV-OC43: 500/FluA-H3: 10/AdvB: 2/RSV-A: 1.5/PIV1: 0.4/FluA: 0.3/hMPV: 0.2.	Respiratory viruses & atypical bacteria	<2 h	PPA 85.1–95.1%; NPA 99.5–99.8%	Multicenter validation	FDA-cleared/CE-IVD	Commercially implemented	NP swab	[38]
Field-Deployable Multiplex PCR	2	10^3^ copies/mL	14 respiratory pathogens	30	PPA 93.6%; NPA 100%	Early clinical validation	Research use	Early field deployment	NP swab	[39]
IoT RT-LAMP	2	SARS-CoV-2: 10^2^ copies/µL for SARS-CoV-2 and 10^2^ pfu/mL for Influenza A and B	SARS-CoV-2, Flu A/B	70	High specificity reported	Pilot study	Research use	Prototype	Respiratory samples	[40]
Passive CRISPR Microfluidic Chip	2	~10 copies/µL	SARS-CoV-2, Flu A/B, HPIV	45	Sensitivity 98.4%; Specificity 100%	Pilot validation	Research use	Prototype	Respiratory samples	[42]
Portable Multiplex RT-PCR	2	10^1^ to 10^2^ copies/reaction	14 respiratory viruses	~60	NR	Laboratory/field testing	Research use	Prototype	NP swab	[43]
HiFi-CAMP	3	SARS-CoV-2: 409 copies/25 μL (statistical LOD) or 3 copies/25 μL (analytical); IAV: 30 copies/25 μL; RSV-A: 30 copies/25 μL	SARS-CoV-2, RSV-A, Influenza A	20–30	Sensitivity 71–90%; Specificity 95.5–100%	Laboratory + clinical validation	Research use	Laboratory-validated	Respiratory samples	[45]
SERS-Nanotag Lateral Flow Microarray	3	0.030–0.041 pM	11 respiratory pathogens	Rapid	NR	Laboratory validation	Research use	Experimental prototype	Respiratory samples	[46]
PEMD Smartphone-ECL	4	10 copies/µL	H1N1, H7N9, Influenza B, Adenovirus	40	Sensitivity 87.5%; Specificity 91.7%	Clinical pilot study	Research use	Early translational prototype	Respiratory samples	[48]
COWFISH2	4	SARS-CoV-2: 0.53–0.63 fM/FluB: 5.20–5.60 fM/FluA: 13.00–17.00 fM	SARS-CoV-2, Flu A/B	20	Sensitivity 94%; Specificity 98%	Proof-of-concept	Research use	Experimental prototype	Respiratory samples	[49]

Abbreviations: LoD, limit of detection; TAT, turnaround time; PPA, positive percent agreement; NPA, negative percent agreement; PPV, positive predictive value; NPV, negative predictive value; NP, nasopharyngeal; BAL, bronchoalveolar lavage; RSV, respiratory syncytial virus; AMR, antimicrobial resistance; NR, not reported. Diagnostic performance values were extracted from the original studies and are not directly comparable because of differences in study design, comparator methods, sample types, and performance metrics. Technology maturity indicates the reported stage of clinical readiness and implementation, ranging from commercially implemented systems to experimental prototypes. Regulatory status reflects approvals or clearances reported in the cited literature and may vary by jurisdiction. Comparisons across tiers should be interpreted in the context of technological maturity, validation level, and intended deployment environment.

## 8. Conclusions and Future Prospectives

The rapid evolution of respiratory point-of-care testing (POCT) technologies has created a highly diverse diagnostic landscape spanning hospital-based molecular systems, portable field-deployable platforms, hardware-lite assays, and digitally connected consumer diagnostics. Rather than viewing these technologies as competing alternatives, the four-tier framework proposed in this review highlights how different POCT architectures address distinct clinical, operational, and public health needs. By classifying platforms according to deployment context, infrastructure dependency, portability, user complexity, and digital integration, this model provides a more practical and implementation-oriented perspective than conventional method-based classifications.

Importantly, the framework emphasizes that successful POCT deployment depends not only on analytical sensitivity and turnaround time, but also on broader considerations including accessibility, cost, reagent stability, scalability, regulatory maturity, and suitability for different healthcare environments. Tier 1 systems currently provide the highest degree of clinical integration and diagnostic accuracy, whereas Tier 2 and Tier 3 platforms improve accessibility in decentralized and resource-limited settings. Tier 4 technologies further extend diagnostics into digitally connected community-based surveillance networks, although many remain in early translational stages.

Despite major advances, several challenges continue to limit widespread implementation of next-generation respiratory POCT systems, including high operational costs, limited multicenter validation, inconsistent regulatory pathways, contamination control, and variability in performance under real-world conditions. Future development should therefore focus on improving assay robustness, affordability, cold-chain independence, multiplex capability, and interoperability with digital health infrastructure. Greater standardization of diagnostic performance reporting and larger multicenter validation studies will also be essential for translating emerging technologies into routine clinical practice.

Overall, the proposed four-tier framework provides a scalable and context-driven strategy for aligning respiratory diagnostic technologies with specific healthcare and epidemiological needs. As molecular diagnostics, microfluidics, biosensing, and digital health technologies continue to converge, future POCT platforms may increasingly integrate features across multiple tiers, enabling more adaptive, decentralized, and equitable approaches to respiratory pathogen detection and outbreak preparedness.

## Data Availability

No new data were created or analyzed in this study. Data sharing is not applicable to this article.

## Abbreviations

Abbreviation	Full Term
AMR	Antimicrobial Resistance
ASSURED	Affordable, Sensitive, Specific, User-friendly, Rapid and Robust, Equipment-free, and Deliverable to End-users
BAL	Bronchoalveolar Lavage
CAMP	Complementary Annealing-Mediated Primer
CE-IVD	Conformité Européenne In Vitro Diagnostic
CMOS	Complementary Metal-Oxide Semiconductor
COVID-19	Coronavirus Disease 2019
CRISPR	Clustered Regularly Interspaced Short Palindromic Repeats
Ct	Cycle Threshold
COWFISH2	Compact Wide-Field Imaging System for High-throughput Femtoliter Analysis 2
DNA	Deoxyribonucleic Acid
ECL	Electrochemiluminescence
FDA	Food and Drug Administration
HCPA	Half-Complementary Primer Amplification
HiFi-CAMP	High-Fidelity Complementary Annealing-Mediated Primer Amplification
hMPV	Human Metapneumovirus
HPIV	Human Parainfluenza Virus
IoT	Internet of Things
LAMP	Loop-Mediated Isothermal Amplification
LoD	Limit of Detection
MERS-CoV	Middle East Respiratory Syndrome Coronavirus
NEAR	Nicking Enzyme Amplification Reaction
NMPA	National Medical Products Administration
NP	Nasopharyngeal
NPA	Negative Percent Agreement
NPV	Negative Predictive Value
PCR	Polymerase Chain Reaction
PEMD	Portable Electrochemical Molecular Diagnostic
POCT	Point-of-Care Testing
PPA	Positive Percent Agreement
PPV	Positive Predictive Value
qPCR	Quantitative Polymerase Chain Reaction
REASSURED	Real-time Connectivity, Ease of Specimen Collection, Affordable, Sensitive, Specific, User-friendly, Rapid and Robust, Equipment-free, and Deliverable to End-users
RNA	Ribonucleic Acid
RPA	Recombinase Polymerase Amplification
RSV	Respiratory Syncytial Virus
RT-LAMP	Reverse Transcription Loop-Mediated Isothermal Amplification
RT-PCR	Reverse Transcription Polymerase Chain Reaction
RT-qPCR	Reverse Transcription Quantitative Polymerase Chain Reaction
SARS-CoV-2	Severe Acute Respiratory Syndrome Coronavirus 2
SERS	Surface-Enhanced Raman Scattering
TAT	Turnaround Time
TCID50	Tissue Culture Infectious Dose 50%
